# Analysis on Drug-Resistance-Associated Mutations among Multidrug-Resistant *Mycobacterium tuberculosis* Isolates in China

**DOI:** 10.3390/antibiotics10111367

**Published:** 2021-11-08

**Authors:** Hongbing Jia, Yuhui Xu, Zhaogang Sun

**Affiliations:** 1 Beijing Key Laboratory in Drug Resistant Tuberculosis Research, Beijing Tuberculosis & Thoracic Tumor Research Institute, Beijing 101149, China; hongbingjia@aliyun.com; 2 Translational Medicine Center, Beijing Chest Hospital, Capital Medical University, Beijing 101149, China; 3 Institute of Chinese Materia Medica, China Academy of Chinese Medical Science, Beijing 100700, China; yuhuixu1234@163.com

**Keywords:** *Mycobacterium tuberculosis*, drug-susceptibility testing, multidrug resistance, mutation

## Abstract

As the causative bacteria of tuberculosis, *Mycobacterium* *tuberculosis (M. tb)* is aggravated by the emergence of its multidrug-resistant isolates in China. Mutations of six of the most frequently reported resistant genes (*rpoB, katG, inhA, embB, gyrA,* and *rpsL*) were detected for rifampicin (RIF), isoniazid (INH), ethambutol (EMB), ofloxacin (OFX), and streptomycin (STR) in this study. The amino acid missense mutations (MMs) and their corresponding single nucleotide polymorphism mutations for all drug-resistant (DR) isolates are described in detail. All isolates were divided into non-extensively drug-resistant (Non-XDR) and preXDR/XDR groups. No statistical differences were detected among MMs and linked MMs (LMs) between the two groups, except for *rpsL* 88 (*p* = 0.037). In the preXDR/XDR group, the occurrence of MMs in *rpoB, katG,* and *inhA* developed phenotypic resistance and MMs of *rpoB* 531, *katG* 315, *rpsL* 43, and *rpsL* 88 could develop high levels of DR. It is necessary to carry out epidemiological investigations of DR gene mutations in the local region, and thus provide necessary data to support the design of new technologies for rapid detection of resistant *M. tb* and the optimization of detection targets.

## 1. Introduction

*Mycobacterium tuberculosis (M. tb)* isolates with multidrug resistance (MDR) are more difficult to treat than drug-susceptible tuberculosis (TB). Close to half a million people developed TB that was resistant to rifampicin worldwide in 2019, and of these, 78% had multidrug-resistant TB (MDR-TB) [1]. The appearance of MDR-TB, including extensively drug-resistant TB (resistance to isoniazid, rifampicin, one fluoroquinolone, and one second-line injectable drug (XDR-TB)) [2], was caused mainly by ineffective treatment, when antibiotics for individual patient are improperly selected or taken, lack of drug-susceptibility testing (DST), and not adopting standard regimens based on the recommendations of the WHO [3,4]. Incorrect diagnosis and treatment of drug-resistant tuberculosis is associated with morbidity, mortality, and ongoing transmission of infection.

As one of the countries with the heaviest burden of MDR-TB, China has reported more and more severe drug-resistant isolates in recent years [1,5]. A baseline survey of drug resistance carried out in 2007 showed that the incidence of multidrug resistance in newly-treated tuberculosis patients and retreated patients was 5.7% and 25.6%, respectively [5].

It is essential to have accurate and rapid diagnosis of MDR-TB, which mainly depends on in vitro testing using either phenotypic methods or molecular techniques [6]. Conventionally, the diagnosis of drug resistance in TB isolates has relied heavily upon culture-based phenotypic DST in liquid or solid media, which has high requirements for TB laboratories, including biosafety conditions, training of technicians, and a long period of weeks to months of incubation. Given the challenges associated with phenotypic DST, rapid molecular tests are increasingly being applied in laboratories, thus providing earlier initiation of appropriate treatment for patients with drug-resistant TB to shorten the regimen.

Drug resistance in the TB complex is mainly conferred through point mutations in specific gene targets, though cell wall structure and the efflux pump also contribute to drug resistance of TB. The reported main amino acid missense mutations (MMs) associated with drug resistance occur in *rpoA, rpoB,* and *rpoC* for rifampicin (RIF); *katG*, *inhA, ahpC,* and *ndh* for isoniazid (INH); *embB* and *embC* for ethambutol (EMB); *rpsL*, *rrs*, *gidB*, *eis*, and *tlyA* for streptomycin (STR); and *gyrA* and *gyrB* for fluoroquinolones (FQs) such as ofloxacin (OFX) [7]. For example, 96% to 100% of RIF-resistant *M. tb* isolates have at least one mutation (the most common mutations are in codons 516, 526, and 531) in the RIF-resistance determining region of *rpoB*, which encodes the RNA polymerase subunit [8]. Of INH-resistant isolates, 42% to 95% have at least one mutation (the most common mutation is in codon 315) in *katG*, which encodes catalase-peroxidase, and 8% to 20% have mutations in the *inhA* open reading frame [9]. Of EMB-resistant isolates, 30% to 67% have at least one mutation in *embB,* which encodes arabinosyltransferase [10,11]. Of STR-resistant isolates, 56% to 72% have mutations in *rpsL*, which encodes ribosomal protein S12 [12,13]. Of FQ-resistant isolates, 50% to 90% have mutations in *gyrA*, which encodes the A subunit of DNA gyrase [14].

Mutation sites of TB drug resistance vary between different countries and regions. A general description of the distribution of these sites is still lacking in China. In this study we analyzed the main mutation sites and frequencies on an amino acid level and base level in order to find the distribution of resistance-associated gene mutations, which will help improve the existing rapid drug-resistance-detection technology and explore new targets for drug activity.

## 2. Materials and Methods

### 2.1. Bacterial Isolates

The isolates involved in this study were all isolated from non-repetitive tuberculosis patients treated at Beijing Chest Hospital in China. In vitro DST for the following drugs, RIF, INH, EMB, OFX, STR, levofloxacin (LVX), capreomycin (CAP), amikacin (AMK), ethionamide (ETO), and p-aminosalicylic acid (PAS) was performed on Löwenstein–Jensen medium following the absolute-concentration method, with 0.2, 40, 2, 2, 10, 2, 40, 30, 40, and 1 g/mL, respectively, as the critical concentrations [15]. Non-XDR, including MDR (defined as isolates resistant to RIF and INH) and MDR plus one drug (EMB, OFX, or STR) resistance, were isolated from 2002 to 2013, including 108 MDR isolates, 209 MDR isolates resistant to EMB, 271 MDR isolates resistant to OFX, and 157 MDR isolates resistant to STR. Forty-six isolates of preXDR/XDR (preXDR is defined as MDR plus resistance to at least four kinds of the drugs (EMB, OFX, STR, LVX, AMK, or ETO) and XDR defined as MDR plus resistance to EMB, OFX, STR, LVX, AMK, ETO, PAS, and CAP), were isolated from 2007 to 2016.

The *M. tb* H37Rv isolate (ATCC 27294) was used as the reference control. Details of all isolates included in Non-XDR and preXDR/XDR groups are listed in Table 1.

### 2.2. Drug Susceptibility Test

Forty-six clinical isolates of preXDR/XDR and *M. tb* H37Rv isolate were tested for minimum inhibitory concentration (MIC) of five anti-TB drugs: RIF, INH, EMB, OFX, STR. Mid-log phase mycobacterial cultures in 7H9 medium were prepared and diluted to OD_600_ 0.02. The diluted bacterial suspension (200 μL) was added to two-fold serially diluted anti-TB drugs (5 μL) in a flat bottom 96–well plate with various concentrations of anti-TB drugs and incubated at 37 °C for one week. Then, 25 μL of 0.02% Resazurin (Sigma) was added to each well and plates were re-incubated for 2 days. A change in color from blue to pink indicated the growth of bacteria. The MIC was defined as the minimum concentration of the anti-TB drugs that prevented the color change. The critical concentrations were 1, 2, 5, 2, and 2 μg/mL for RIF, INH, EMB, OFX, and STR, respectively [15]. To control this effect in this study, a positive control, negative control, and the MIC test for each tested isolate were employed twice in parallel in the study.

### 2.3. DNA Extraction

All the selected clinical isolates were subcultured twice on Löwenstein–Jensen medium from the *M. tb* stock solutions stored at −70 °C in small aliquots. DNA extraction was carried out by QIAamp DNA mini Kit (QIAGEN, Hilden, Germany). The isolates were removed from the slants of culture medium with an inoculation loop and suspended in 180 μL of Buffer ATL by vigorous stirring. The bacterial pellet was collected after centrifugation for 10 min at 7500 rpm and suspended in 180 μL of the enzyme solution (20 mg/mL lysozyme; 20 mM Tris·HCl, pH 8.0; 2 mM EDTA; 1.2% Triton). The mixture was incubated for 30 min at 37 °C, then added with 20 μL proteinase K and 200 μL buffer AL and mixed by vortexing. After incubation at 56 °C for 30 min, the remaining steps followed the “Protocol: DNA Purification from Tissues“ from step 6. Finally, the supernatant was transferred to another 1.5 mL tube and preserved at −20 °C until further use for polymerase chain reaction (PCR). 

### 2.4. Drug-Resistance-Related Genes and DNA Sequencing

The six most frequently reported resistant genes detected for RIF, INH, EMB, OFX, and STR were *rpoB, katG, inhA, embB, gyrA,* and *rpsL*) were. PCR primers of target genes are listed in Table S1 in Supplementary Materials. The primers (from Sangon Biotech, Beijing, China) and the genomic DNA was used as the template to perform PCR amplification as follows. Each PCR mixture was prepared in a volume of 50 μL containing 25 μL of 2 × PCR mixture, 2 μL of DNA template, and 0.2 μM each primer set. PCR was done under the following conditions: initial denaturation at 95 °C for 5 min and then 35 cycles of denaturation at 94 °C for 30 s, annealing at 65 °C for 30 s, and extension at 72 °C for 30 s, followed by a final extension at 72 °C for 10 min. The PCR products were purified and sequenced by Sangon Biotech (Shanghai, China). Mutations were identified using BLAST and compared with those of *M. tb* H37Rv isolate using the ClustalW multiple sequence alignment. The impacts of MMs on protein structure were indicated by SMART (Simple Modular Architecture Research Tool) and AlphaFold [16].

### 2.5. Statistical Analysis

The data collected were compiled and analyzed with SPSS statistical software version 24.0 (IBM, New York, NY, USA). The chi-square test or Fisher′s exact test was used to compare the two groups of data. When the sample size of the two groups was greater than 40, the chi-square test was performed; when the sample size of one group was less than 40, the fisher exact test was used. The significance threshold was set to 0.05.

## 3. Results

### 3.1. Amino Acid Missense Mutation Types and Their Corresponding SNP Mutation Types for All Drug-Resistant Isolates

**Figure 1:**
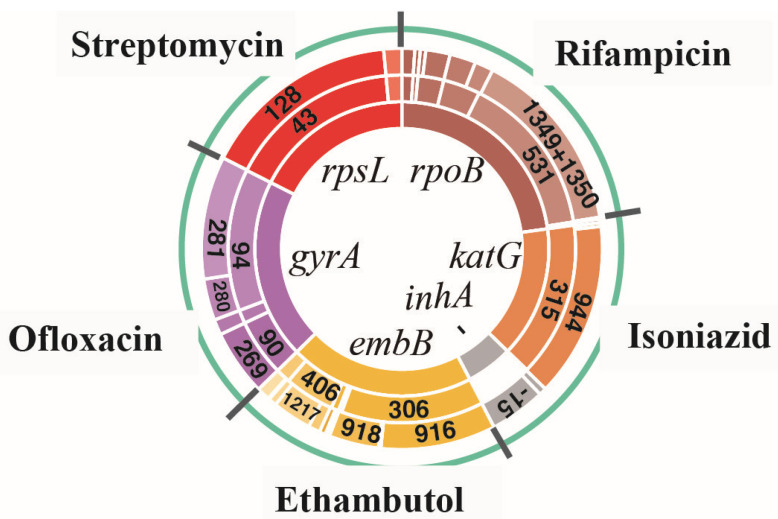
Proportion of amino acid missense mutation types and their corresponding SNP mutation types for all drug-resistant isolates. The innermost circle represents the six most frequently reported resistant genes (*rpoB, katG, inhA*, *embB, gyrA,* and *rpsL*); the middle circle represents the MMs corresponding to each type of resistant gene, respectively; and the outermost circle represents the SMs corresponding to each MM, respectively.

**Figure 2:**
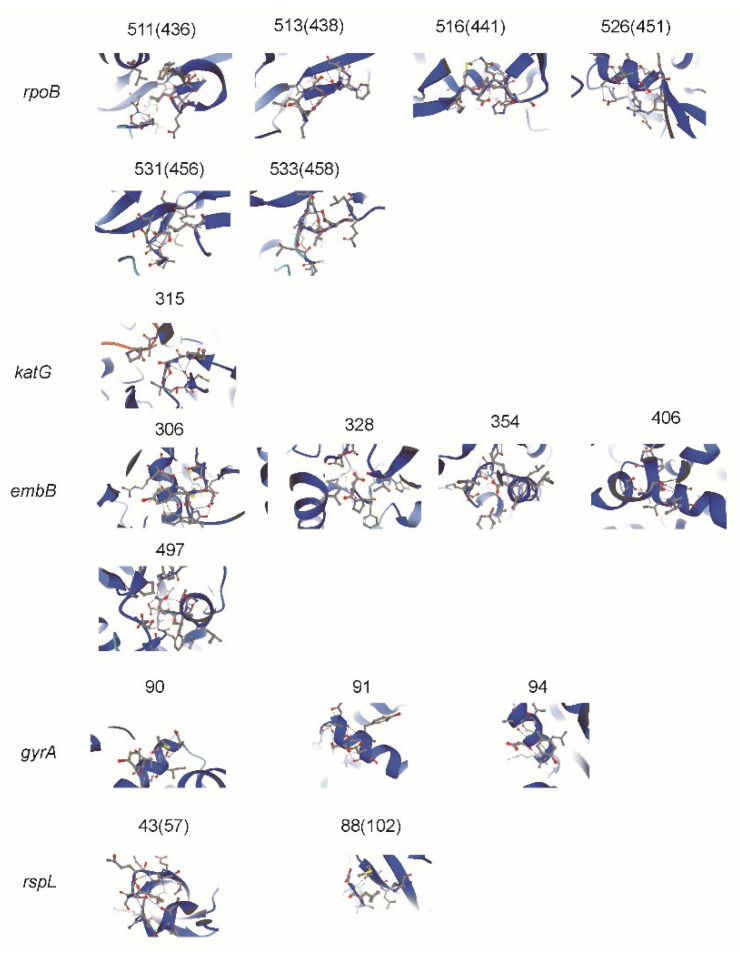
Impacts of amino acid substitutions of missense mutation was predicted by Alphafold. The mutations sites are marked with the shadows. The missense mutation sites (corresponding sites in Alphafold) were marked at the top of protein structures.

For all drug-resistant isolates, the most common types of resistance-related genes (*rpoB, katG, inhA, embB, gyrA,* and *rpsL*) were analyzed for the incidence of MMs and the corresponding single nucleotide polymorphism mutations (SMs). The main results can be seen in Figure 1. The details can be found in Table S2 in Supplementary Materials. The predicted impacts of MM substitutions for protein function are shown in Figure 2.

Among 154 RIF-resistant isolates (108 MDR and 46 preXDR/XDR isolates), *rpoB* gene mutations were detected in 144 isolates (93.5%, 144/154). The most common MM occurred at codon 531, a total of 101 isolates (65.6%, 101/154), of which 90 isolates (58.4%, 90/154) had an MM in Ser531Leu with corresponding SMs in C1349T and C1350G; of which there were 10 isolates (6.5%, 10/154) in Ser531Trp with SMs in C1349G and C1350G and one isolate (0.6%, 1/154) in Ser531Tyr with an SM in C1349A. The second most common MM occurred at codon 526 (His). A total of 23 isolates (14.9%, 23/154) showed an MM at this position, of which there were 14 isolates (9.1%, 14/154) with an SM in base 1333 and 10 isolates (6.5%, 10/154) with an SM in base 1334. In addition, 16 isolates (10.4%, 16/154) had an MM at codon 516, of which there were three isolates (1.9%, 3/154) with an SM in base 1303 and 13 isolates (8.4%, 13/154)) with an SM in base 1304; seven isolates (4.5%, 7/154) showed an MM at codon 511 (Leu511Pro) with an SM in T1289C; three isolates (1.9%, 3/154) showed an MM at codon 513 (Gln513Lys) with an SM in C1294A; and two isolates (1.3%, 2/154) showed an MM at codon 533 (Leu533Pro) with an SM in T1355C.

Among the 154 INH-resistant isolates (108 MDR and 46 preXDR/XDR isolates), gene mutations on *katG* (62.3%, 96/154) and *inhA* (22.1%, 34/154) were detected in 122 isolates (79.2%, 122/154). The most common MM occurred at codon 315 of KatG: 90 isolates (58.4%, 90/154) showed an MM in Ser315Thr with an SM in G944C, four isolates (2.6%, 4/154) showed MM in Ser315Asn with SM in G944A, and two isolates (1.3%, 2/154) showed an MM in Ser315Gly with an SM in A943G. An SM of the *inhA* promoter region occurred at base 15 (C-T) in 30 isolates (19.5%, 30/154), at base -8 (T-C) in three isolates (1.9%, 3/15) and at base -8 (T-G) in one isolate (0.6%, 1/154).

Among the 241 EMB-resistant isolates (209 MDR and 32 preXDR/XDR isolates), 187 isolates (77.6%, 187/241) contained *embB* gene mutations. The most common MM occurred at codon 306, a total of 117 isolates (48.5%, 117/241), including an MM in Met306Val of 90 isolates (37.3%, 90/241) with an SM in A916G, an MM in Met306Ile of 41 isolates (12.9%, 41/241) with an SM in G918A/C/T and an MM in Met306Leu of six isolates (2.5%, 6/241) with an SM in A916C/T. The second most common MM occurred at codon 406, 41 isolates (17.0%, 41/241) in total, including an MM mainly in Gly406Ala of 16 isolates (6.6%, 16/241) with an SM in G1217C of 12 isolates (5.0%, 12/241) with an SM in G1217A. In addition, 12 isolates (5.0%, 12/241) had an MM at codon 497, of which there were mostly MMs in Gln497Arg of 11 isolates with an SM in A1490G; six isolates (2.5%, 6/241) had an MM at codon 354, of which there were mostly MMs in Asp354Ala of five isolates with an SM in A1060C; and three isolates (1.2%, 3/241) had an MM at codon 328, of which there was one isolate each with Asp328His, Asp328Tyr, and Asp328Gly, in G982C, G982T, and A983T, respectively.

Among the 296 OFX-resistant isolates (271 MDR and 25 preXDR/XDR isolates), 202 isolates (68.2%, 202/296) had gene mutations in *gyrA*. The most common MM occurred at codon 94, a total of 169 isolates (57.1%, 169/296), including an MM mainly in Asp94Gly of 83 isolates (28.0%, 83/296) with SMs in A281G and in Asp94Ala of 43 isolates (14.5%, 43/296) and SMs in A281C and in Asp94Asn of 32 isolates (10.8%, 32/296), and an SM in G280A. The second most common MM occurred at codon 90, all the 68 isolates (17.0%, 41/241) had an MM in Ala90Val with an SM in C269T. In addition, 16 isolates (5.4%, 16/296) had an MM at codon 91 (Ser91Pro) with an SM inT271C.

Among 190 isolates of STR-resistant isolates (157 MDR and 33 preXDR/XDR isolates), 144 isolates (75.8%, 144/190) had genetic mutations in *rpsL*. The most common MM occurred at codon 43, a total of 132 isolates (69.5%, 132/190), including an MM in Leu43Ala with an SM in A128G. MMs of the rest of the 12 isolates (6.3%, 12/190) occurred at codon 88 (Leu88Ala) with an SM in A263G. 

### 3.2. Analysis of Linked Missense Mutations Found in All Drug-Resistant Isolates 

In the analysis of Non-XDR and preXDR/XDR groups, it was found that in addition to STR-resistant isolates, some other drug-resistant isolates usually have linked missense mutations (LMs) with two MMs always happening at the same time. LMs occurred most frequently in OFX-resistant isolates, followed by EMB-resistant, INH-resistant, and RIF-resistant isolates. There were 50 isolates with LMs (24.8%, 50/202) in OFX-resistant isolates, of which 40 isolates with LMs (*gyrA* 90 + *gyrA* 94) and 10 isolates with LMs (*gyrA* 90 + *gyrA* 91) were found; 11 isolates with LMs (5.9%, 11/187) in EMB-resistant isolates, of which six isolates with LMs (*embB* 306 + *embB* 406), two isolates with LMs (*embB* 306 + *embB* 328), two isolates with LMs (*embB* 306 + *embB* 354), and one isolate with LMs (*embB* 306 + *embB* 497) were found; seven isolates with LMs (5.7%, 7/123) in INH-resistant isolates, all of which had LMs (*katG* 315 + *inhA*); eight isolates with LMs (3.5%, 8/144) in RIF-resistant isolates, of which four isolates with LMs (*rpoB* 511 + *rpoB* 516), one isolate with LMs (*rpoB* 511 + *rpoB* 526), one isolate with LMs (*rpoB* 516 + *rpoB* 526), one isolate with LMs (*rpoB* 526 + *rpoB* 531), and one isolate with LMs (*rpoB* 516 + *rpoB* 531) were found. By analyzing the corresponding MIC of these DR isolates with LMs, we found that there was no obvious high-level DR. There was no significant difference in the incidence of LMs between the Non-XDR and preXDR/XDR groups (Table 2).

### 3.3. Comparison for the Occurrence of Amino Acid Missense Mutation Types between Non-XDR and preXDR/XDR Groups 

**Figure 3:**
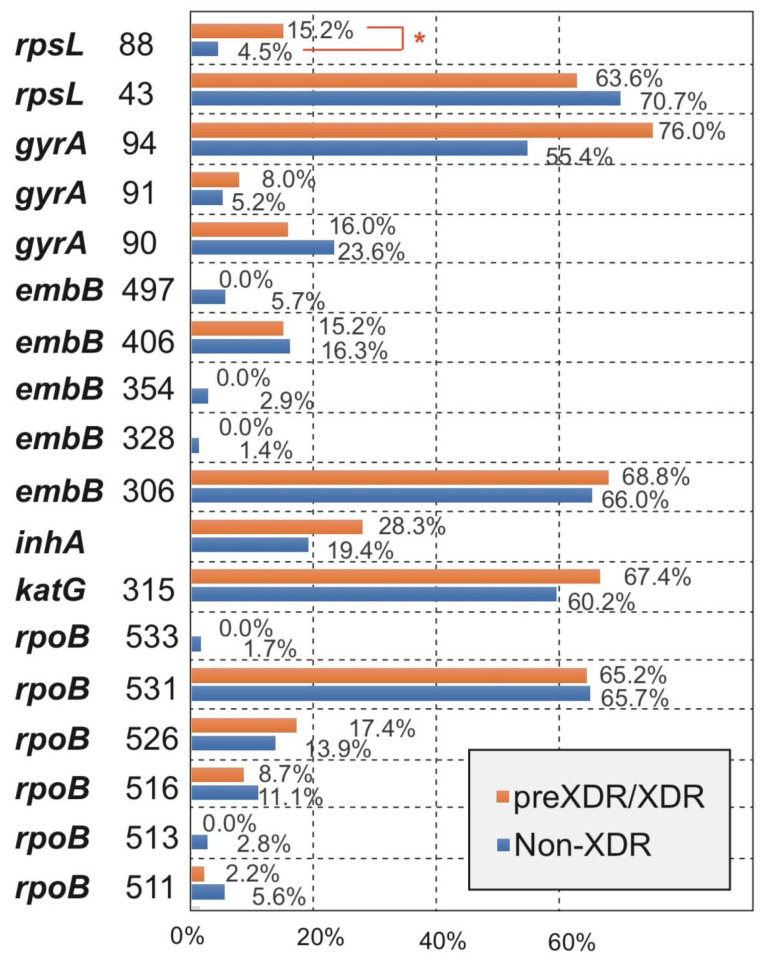
Comparison for the occurrence of amino acid missense mutation types between Non-XDR and preXDR/XDR groups. *, *p*-value < 0.05 was considered significant.

By analyzing the differences in the main MM types of the five drugs between the Non-XDR and preXDR/XDR groups, we found that all MM types of the other four drugs were not significantly different except for streptomycin (Figure 3). Among the two MM types of streptomycin, *rpsL* 43 was not significantly different, and *rpsL* 88 was significantly different (*p* < 0.05) between the two groups. The details can be found in Table S3 in Supplementary Materials.

### 3.4. Correspondence between MM Type and MIC Value in preXDR/XDR Isolates

**Figure 4:**
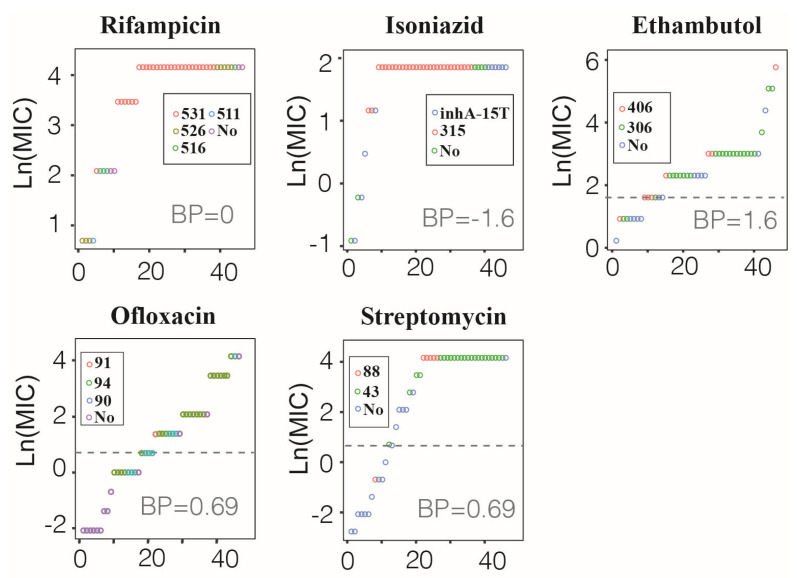
Correspondence between amino acid missense mutation types and MIC value in preXDR/XDR isolates. The *X*-axis represents the cumulative number of isolates, the *Y*-axis r represents the natural logarithm of the MIC value, the dotted line represents the breakpoint of drug resistance. BP, the natural logarithm of breakpoint MIC value; No, not detected MM.

All 46 preXDR/XDR isolates had 27 preXDR and 19 XDR, of which all isolates were resistant to rifampicin and isoniazid, 32 isolates were EMB-resistant, 25 isolates were OFX-resistant, and 33 isolates were STR-resistant (Table 3). The distribution of MIC values corresponding to different MM types is shown in Figure 4.

Forty-two RIF-resistant isolates (91.3%, 42/46) were detected as *rpoB* gene mutations, of which 29 isolates (63.0%, 29/46) with MM (*rpoB* 531) had MIC distribution between 64 μg/mL and 8 μg/mL (64 μg/mL (79.%, 22/29), 32 μg/mL (20.7%, 6/29), and 8 μg/mL (3.4%, 1/29)); eight isolates (17.3%, 8/46) with MM (*rpoB* 526) had MIC distribution between 64 μg/mL and 2 μg/mL (64 μg/mL (62.5%, 5/8) and 2 μg/mL (37.5%, 3/8)). The remaining four isolates with MM (*rpoB* 516) and one isolate with MM (*rpoB* 511) had MIC distribution between 64 μg/mL and 2 μg/mL. Twenty-eight isolates (66.7%, 28/42) with MM (*rpoB* 531) developed high-level drug resistance (32 μg/mL–64 μg/mL). The MICs of four isolates with *rpoB* MM(−) ranged from 64 μg/mL to 8 μg/mL. 

Forty INH-resistant isolates (87.0%, 40/46) were detected as *katG* and *inhA* gene mutations, of which 30 isolates (75.0%, 30/40) with MM (*katG* 315) had MIC distribution between 6.4 μg/mL and 3.2 μg/mL (6.4 μg/mL (93.3%, 28/30) and 3.2 μg/mL (6.7%, 2/30)) and 10 isolates (25.0%, 10/40) with *inhA*-15T had MIC distribution between 6.4 μg/mL and 0.4 μg/mL. Twenty-eight isolates (70.0%, 28/40) with MM (*katG* 315) developed high-level drug resistance (3.2 μg/mL–6.4 μg/mL). The MICs of six isolates with *katG*/*inhA* MM(−) ranged from 64 μg/mL to 8 μg/mL.

Thirty-three EMB-resistant isolates (71.7%, 33/46) were detected as *embB* gene mutations, of which 25 isolates (75.8%, 25/33) with MM (*embB* 306) had MIC distribution between 160 μg/mL and 2.5 μg/mL (160 μg/mL (8.0%, 2/25), 40 μg/mL (2.5%, 1/25), 20 μg/mL (48.0%, 12/25), 10 μg/mL (28.0%, 7/25), 5 μg/mL (2.5%, 1/25), and 2.5 μg/mL (5.0%, 2/25)); and eight isolates (24.2%, 8/33) with MM (*embB* 406) had MIC distribution between 320 μg/mL and 2.5 μg/mL. Only three isolates (9.1%, 3/33) with MM (*embB* 306) or MM (*embB* 406) developed high-level drug resistance (80 μg/mL–320 μg/mL). The MICs of 13 isolates with *embB* MM(−) ranged from 80 μg/mL to 1.25 μg/mL.

Thirty-three OFX-resistant isolates (71.7%, 33/46) were detected as *gyrA* gene mutations, of which 22 isolates (66.7%, 22/33) with MM (*gyrA* 94) had MIC distribution between 64 μg/mL and 1 μg/mL (64 μg/mL (4.5%, 1/22), 32 μg/mL (27.2%, 6/22), 8 μg/mL (31.8%, 7/22), 4 μg/mL (13.6%, 3/22), 2 μg/mL (4.5%, 1/22), and 1 μg/mL (4.5%, 1/22)) and 10 isolates (30.3%, 10/33) with MM (*gyrA* 90) had MIC distribution between 64 μg/mL and 1 μg/mL; the MIC of one isolate (30.3%, 1/33) with MM (*gyrA* 91) was 4 μg/mL. Only eight isolates (24.2%, 8/33) with MM (*gyrA* 94) or MM (*gyrA* 90) developed high-level drug resistance (32 μg/mL–64 μg/mL). The MICs of 13 isolates with *gyrA* MM(−) ranged from 64 μg/mL to 0.125 μg/mL.

Twenty-nine STR-resistant isolates (63.0%, 29/46) were detected as *rpsL* gene mutations, of which 23 isolates (79.3%, 23/29) with MM (*rpsL* 43) had MIC distribution between 64 μg/mL and 2 μg/mL (64 μg/mL (82.6%, 19/23), 32 μg/mL (8.7%, 2/23), 16 μg/mL (4.3%, 1/23), and 2 μg/mL (4.3%, 1/23)) and six isolates (34.5%, 10/29) with MM (*rpsL* 88) had MIC distribution between 64 μg/mL–0.5 μg/mL (64 μg/mL (83.3%, 5/6) and 0.5 μg/mL (16.7%, 1/6). Twenty-six isolates (89.7%, 26/29) with MM (*rpsL* 43) or MM (*rpsL* 88) developed high-level drug resistance (32 μg/mL–64 μg/mL). The MICs of 17 isolates with *rpsL* MM(−) ranged from 64 μg/mL to 0.0625 μg/mL.

## 4. Discussion

Drug resistance of first/second-line anti-tuberculosis drugs (RIF, INH, EMB, OFX, and STR) was significantly related to the reported mutations of gene locus (*rpoB, katG, inhA, embB, gyrA,* and *rpsL*) in *M. tb*, but there were still differences in the location and frequency of these gene mutations in different regions. Our study focused on analyzing the genetic mutation characteristics of multidrug-resistant *M. tb* in China and found that all *rpoB* MMs of RIF-resistant isolates occurred in “hot spots”, 81 base regions of the *rpoB* gene (codons 507–533), of which codon 531 (58.4%) was the most common, followed by codons 526 and 516, and the three types accounted for about 83.7%. All *katG* MMs of INH-resistant isolates occurred in codon 315, *inhA* mutations were mainly -15C/T, and the two types accounted for about 79.9%; *embB* MMs of EMB-resistant isolates occurred in codon 306 (48.5%), which was the most common, followed by codon 406, and the two types accounted for about 65.5%; *gyrA* MMs of OFX-resistant isolates were the most common at codon 94 (57.1%), followed by codon 90, and the two types accounted for about 74.1%; *rpsL* MMs of STR-resistant isolates were the most common at codon 43 (69.5%), and the rest were at codon 88. The mutations affected the protein function for the drug activities by the changes of secondary and 3D structure [17,18]. In the preXDR/XDR group, all 42 isolate-detected MMs of *rpoB, katG,* and *inhA* developed rifampicin and isoniazid resistance and MMs of *rpoB* 531, *katG* 315, *rpsL* 43 and *rpsL* 88 could develop high levels of drug resistance; the same results were reported in [12,19,20]. 

Among the resistant isolates in this study, the MM frequencies of *rpoB*, *katG*, *embB*, *gyrA,* and *rpsL* genes were similar to the reported data in China [13,14]; but there were still differences between geographical regions [21]—such as codon 531 of *rpoB*, 58.4% in this study, 80.9% in Kazakhstan [22], 52.4% in Brazil [23], 52% in Australia [24], 41.5% in Russia [25], and 31% in Hungary [26]. This was mainly related to the transmission route and evolution of tuberculosis in different locations. Comparing the MM frequency of five kinds of DR isolates between non-XDR and preXDR/XDR groups, it is found that only *rpsL* 88 had a significant difference. This result could have been caused by the small number of preXDR/XDR included isolates, or an increase in the mutation rate of *rpsL* 88 developed by preXDR/XDR, which requires further research to verify. Although the occurrence rate (76.0%) of *gyrA* 94 in the preXDR/XDR group was more higher than that (55.4%) in the XDR group, there were no statistical differences (*p* = 0.057). Interestingly, it was found that the occurrence rates of *rpoB* 511/513/516/533, *rpsL* 43, *gyrA* 90, and *embB* 328/354/496/497 in the non-XDR group were higher in preXDR/XDR group, which was most likely caused by the small number of isolates in the XDR group. LMs were found in four types of DR (RIF, INH, EMB, and OFX) isolates. In particular, OFX LMs (*gyrA* 90 + *gyrA* 94) were as high as 26.7%, but OFX-resistant isolates with these LMs didn′t develop the high level MIC. In comparison of mutations in *gyrA* only and of ofloxacin-resistant strains with mutations in both *gyrA* and *gyrB* genes, the difference in MICs was not statistically significant [12].

At present, the common application technologies for rapid detection of resistant *M. tb* mainly include real-time fluorescent PCR, gene chip technology, and high-throughput gene sequencing technology. While analyzing the types and frequencies of various codon mutations, this study further analyzed the base mutation sites and frequencies corresponding to each codon mutation to provide necessary data support for the future design and optimization of DR molecular testing in TB. For example, RIF-resistant isolates had 100% *rpoB* MMs, and codon 531 accounted for 65.6%, of which MMs corresponded to three types of SM—Ser531Leu with C1349T and C1350G, Ser531Trp with C1349G and C1350G, and Ser531Tyr with C1349A. Therefore, when detecting MMs in codon 531 by the molecular techniques, only the base 1349 could be determined.

There were some shortcomings in our research. For the main purpose of this study, which was to screen the DR gene mutation patterns of resistant clinical *M. tb*, the drug-sensitive isolates were not detected, although some MMs were also found in phenotype-sensitive isolates [6,8,11,15,26]. The screening of DR isolates only focused on the reported main genes which were significantly related to resistant *M. tb*, and some new DR gene analysis was not included. Moreover, the isolates included in the study were mainly MDR and XDR, and there was a lack of analysis between different types of resistant clinical isolates, such as single-resistant, MDR, preXDR, and XDR ones. Furthermore, the isolates included in this study spanned more than 10 years, and the drug resistance and susceptibility rates of *M. tb* for each year were not available.

## 5. Conclusions

The effective management of MDR-TB relies on the rapid diagnosis and treatment of drug-resistant infections. Early detection of DR tuberculosis is a key factor in reducing and controlling the spread of these DR strains. It is necessary to carry out epidemiological investigations of DR gene mutations in the region, and provide necessary data support for the design of new technologies for rapid detection of resistant *M. tb* and the optimization of detection targets.

## Figures and Tables

**Table 1:** Details of all isolates included in Non-XDR and preXDR/XDR groups.

Isolate Name		INH	RIF	EMB	OFX	STR
Non-XDR	DR	108	108	209	271	157
	DR and MM(+)	102	82	161	180	117
preXDR/XDR	DR	46	46	32	25	33
	DR and MM(+)	42	40	26	22	27

DR, drug-resistant; MM, missense mutation; INH, isoniazid; RIF, rifampin; EMB, ethambutol; OFX, ofloxacin; STR, streptomycin, MM(+), detected MM.

**Table 2:** Comparison for the incidence of linked amino acid missense mutations between the Non-XDR and preXDR/XDR groups.

Linked Missense Mutations	Incidence Rate % (No./Total AM No.*)		*p* Value
	Non-XDR	preXDR/XDR	
Rifampicin	6.9% (7/102)	2.4% (1/42)	0.505
*rpoB* 511 + *rpoB* 516	3.9% (4/102)	/	/
*rpoB* 511 + *rpoB* 526	1.0% (1/102)	/	/
*rpoB* 516 + *rpoB* 526	1.0% (1/102)	/	/
*rpoB* 526 + *rpoB* 531	1.0% (1/102)	/	/
*rpoB* 516 + *rpoB* 531	/	2.4% (1/42)	/
Isoniazid	4.9% (4/82)	7.5% (3/40)	0.891
*katG* 315 + *inhA*	4.9% (4/82)	7.5% (3/40)	0.891
Ethambutol	6.2% (10/161)	3.8% (1/26)	1.000
*embB* 306 + *embB* 328	1.2% (2/161)	/	/
*embB* 306 + *embB* 354	1.2% (2/161)	/	/
*embB* 306 + *embB* 406	3.1% (5/161)	3.8% (1/26)	1.000
*embB* 306 + *embB* 497	0.6% (1/161)	/	/
Ofloxacin	26.7% (48/180)	9.1% (2/22)	0.072
*gyrA* 90 + *gyrA* 91	5.0% (9/180)	4.5% (1/22)	1.000
*gyrA* 90 + *gyrA* 94	21.7% (39/180)	4.5% (1/22)	0.053

* The total number of isolates with MMs when each drug was resistant; *p*-value < 0.05 was considered significant.

**Table 3:** The details of drug resistance and amino acid missense mutations in 46 preXDR/XDR isolates.

Isolate No.		INH	RIF	EMB	OFX	STR
Total DR	DR and MM(+)	40	42	26	22	27
	DR and MM(−)	6	4	6	3	6
	Total no.	46	46	32	25	33
Total DS	DS and MM(+)	0	0	7	11	2
	DS and MM(−)	0	0	7	10	11
	Total no.	0	0	14	21	13

DR, drug-resistant; DS, drug-sensitive; MM, missense mutation; INH, isoniazid; RIF, rifampin; EMB, ethambutol; OFX, ofloxacin; STR, streptomycin; MM(+), detected MM; MM(−), not detected MM.

## Data Availability

The data presented in this study are available in supplementary materials here.

## Supplementary Materials

The following are available online at https://www.mdpi.com/article/10.3390/antibiotics10111367/s1, Table S1: Primers used in this study, Table S2: The incidence of amino acid missense mutation and their corresponding SNP mutation for five drugs in all drug-resistant strains (including Non-XDR and preXDR/XDR), Table S3: The details for the Occurrence of Amino Acid Missense Mutation Types between Non-XDR and preXDR/XDR Groups.
