# Supplementary material for: Analysis on Drug-Resistance-Associated Mutations among Multidrug-Resistant Mycobacterium tuberculosis Isolates in China
Source: Antibiotics (Basel). 2021 Nov 8;10(11):1367. doi: 10.3390/antibiotics10111367 (PMC8614678; doi:10.3390/antibiotics10111367)
Supplement: Supplementary file 1 [file antibiotics-10-01367-s001.zip › antibiotics-1436771-supplementary.pdf]

antibiotics

MDPI

Article

Analysis on drug resistance-associated mutations among multi-drug-resistant *Mycobacterium tuberculosis* isolates in China

Hongbing Jia[1,2], Yuhui Xu[3], Zhaogang Sun[1,2,*]

1 Beijing Key Laboratory in Drug Resistant Tuberculosis Research, Beijing Tuberculosis & Thoracic Tumor Research Institute, Beijing, 101149, China.
2 Translational Medicine Center, Beijing Chest Hospital, Capital Medical University, Beijing, 101149, China.
3 Institute of Chinese Materia Medica, China Academy of Chinese Medical Science, Beijing, 100700, China
* **Correspondence:** author: sunzg75@163.com; Tel./Fax: 86-10-89509161

Supplementary Materials

Table S1. Primers used in this study.

Name	5'-3'	Size of PCR product (bp)	Location of PCR product
rpoB-F1	TAGTTGCGTGCGTGAGATCC	601	75-656
rpoB-R1	TCTCGGTCATCATCGGGAAG		
rpoB-F2	CGGCTCCACTGTTCGTCACC	574	577-1131
rpoB-R2	CCGAGCTTCTTGTTGACCTT		
rpoB-F3	AGCCCCCGACCAAAGAGTCA	557	1042-1582
rpoB-R3	CAGCCCGGCACGCTCAC		
rpoB-F4	CGCTGTCGGGGTTGACCCAC	512	1522-2014
rpoB-R4	CGCCTGGCGCTGCATGTTTG		
rpoB-F5	GGACTACATGGACGTCTCGC	612	1916-2509
rpoB-R5	ACCTCGTCGGAGATGTTCG		
rpoB-F6	TGTACTGACGACGGCGAGAT	571	2307-2860
rpoB-R6	TGCCGATCACGCCCTTGT		
rpoB-F7	CCGCGAGGACGAGGACGAGT	577	2750-3307
rpoB-R7	GGTGGAGCGGGCGTGGATCT		
rpoB-F8	GGCGAGCCGTTCCCGTACCC	594	3234-3808
rpoB-R8	TGTCCTCCGCGGTAGCAAGA		
KatG-F1	ACGAGGCGGAGGTCATCTAC	667	179-846
KatG-R1	CGAAGCCGAACCCGAACGTC		
KatG-F2	GCTGCTGTGGCCGGTCAAGA	635	727-1342
KatG-R2	CGTCCTTGGCGGTGTATTGC		
KatG-F3	GGACGAACACCCCCGACGAAA	635	1251-1886

KatG-R3	CGCCGCGGAGTTGAATGACT		
KatG-F4	TCGGGTGGGAGGTCAACGAC	533	1797-2330
KatG-R4	GCCCTGGTAGGTCCCGTCA		
KatG-F5	TGCTTACGCTCAGTGCCCCT	541	2136-2677
KatG-R5	CTCATCCCCGTCTCGTCATC		
mabA/inhA-F1	TCGACGGCGGCATGGGTAT		
mabA/inhA-R1	ACCGACTCCAACGCGCTCTT	559	155-694
mabA/inhA-F2	CATCCACATCTCGGCGTATT	550	558-1089
mabA/inhA-R2	GGCCCCGGGTAACGTTCTC		
embB-F	ATTCGGCTTCCTGCTCTGG		
embB-R	CACACCGTAGCTGGAGACAT	1112	851-1962
gyrA-F	CAGCTACATCGACTATGCGA	320	78-398
gyrA-R	CGGGCTTCGGTGTACCTCAT		
rpsL-F	CCAGCAGCTGGTCCGCAAGG	373	12-ORF
rpsL-R	TGCGTGGCATCAGCCCTTCTC		

Table S2. The incidence of amino acid missense mutation and their corresponding SNP mutation for five drugs in all drug-resistant strains (including Non-XDR and preXDR/XDR).

**Drug**		**Nucleotide**	
**missense mutation**	**Incidence rate % (no./total no.*)**	**SNP mutation**	**Incidence rate % (no./total no.*)**
**Rifampicin**			
rpoB 511	4.5% (7/154)		
Leu511Pro(CTG-CCG)	4.5% (7/154)	T1289C	4.5% (7/154)
rpoB 513	1.9% (3/154)		
Gln513Lys(CAA-AAA)	1.9% (3/154)	C1294A	1.9% (3/154)
rpoB 516	10.4% (16/154)		
Asp516Tyr(GAC-TAC)	1.9% (3/154)	G1303T	1.9% (3/154)
Asp516Ala(GAC-GCC)	1.3% (2/154)	A1304C	
Asp516Val(GAC-GTC)	4.5% (7/154)	A1304T	8.4% (13/154)
Asp516Gly(GAC-GGC)	2.6% (4/154)	A1304G	
rpoB 526	14.9% (23/154)		
His526Asp(CAC-GAC)	3.9% (6/154)	C1333G	
His526Tyr(CAC-TAC)	3.9% (6/154)	C1333T	9.1% (14/154)
His526Asn(CAC-AAC)	0.6% (1/154)	C1333A	
His526Leu(CAC-CTC)	3.2% (5/154)	A1334T	
His526Arg(CAC-CGC)	2.6% (4/154)	A1334G	6.5% (10/154)
His526Gly(CAC-GGC)	0.6% (1/154)	C1333G+A1334G[#]	
rpoB 531	65.6% (101/154)		
Ser531Leu(TCC-TTG)	58.4% (90/154)	C1349T+C1350G[#]	64.9% (100/154)
Ser531Trp(TCC-TGG)	6.5% (10/154)	C1349G+C1350G[#]	
Ser531Tyr(TCC-TAC)	0.6% (1/154)	C1349A	0.6% (1/154)
rpoB 533	1.3% (2/154)		
Leu533Pro(CTG-CCG)	1.3% (2/154)	T1355C	1.3% (2/154)

**Isoniazid**			
katG 315	62.3% (96/154)		
Ser315Gly(AGC-GGC)	1.3% (2/154)	A943G	1.3% (2/154)
Ser315Asn(AGC-AAC)	2.6% (4/154)	G944A	61.0% (94/154)
Ser315Thr(AGC-ACC)	58.4% (90/154)	G944C	
inhA	22.1% (34/154)		
		-8(T-G)	0.6% (1/154)
		-8(T-C)	1.9% (3/154)
		-15(C-T)	19.5% (30/154)
**Ethambutol**			
embB 306	48.5% (117/241)		
Met306Leu(ATG-CTG)	2.5% (6/241)	A916C	40.2% (97/241)
Met306Leu(ATG-TTG)		A916T	
Met306Val(ATG-GTG)	37.3% (90/241)	A916G	
Met306Ile(ATG-ATA)	12.9% (41/241)	G918A	17.4% (42/241)
Met306Ile(ATG-ATC)		G918C	
Met306Ile(ATG-ATT)		G918T	
Met306Arg(ATG-CCT)	0.4% (1/241)	A916C+T917C+G918T #	0.8% (2/241)
Met306Lys(ATG-AAG)	0.4% (1/241)	T917A	
embB 328	1.2% (3/241)		
Asp328His(GAT-CAT)	0.4% (1/241)	G982C	0.8% (2/241)
Asp328Tyr(GAT-TAT)	0.4% (1/241)	G982T	
Asp328Gly(GAT-GGT)	0.4% (1/241)	A983T	0.4% (1/241)
embB 354	2.5% (6/241)		
Asp354Asn(GAC-AAC)	0.4% (1/241)	G1059A	0.4% (1/241)
Asp354Ala(GAC-GCC)	2.1% (5/241)	A1060C	2.1% (5/241)
embB 406	17.0% (41/241)		
Gly406Cys(GGC-TGC)	1.2% (3/241)	G1216T	4.1% (10/241)
		G1216A	
		G1216C	
Gly406Ala(GGC-GCC)	6.6% (16/241)	G1217C	13.7% (33/241)
Gly406Asp(GGC-GAC)	5.0% (12/241)	G1217A	
Gly406Arg(GGC-AGG)	0.8% (2/241)	G1216A+C1218G #	2.9% (7/241)
Gly406Lys(GGC-AAA)	0.8% (2/241)	G1216A+G1217A+C1218A #	
Gly406Pro(GGC-CCG)	1.2% (3/241)	G1216C+G1217C+C1218G #	
embB 497	5.0% (12/241)		
Gln497Lys(CAG-AAG)	0.4% (1/241)	C1489A	0.4% (1/241)
Gln497Arg(CAG-CGG)	4.6% (11/241)	A1490G	4.6% (11/241)
**Ofloxacin**			
gyrA 90	23.0% (68/296)		
Ala90Val(GCG-GTG)	23.0% (68/296)	C269T	23.0% (68/296)
gyrA 91	5.4% (16/296)		
Ser91Pro(TCG-CCG)	5.4% (16/296)	T271C	5.4% (16/296)
gyrA 94	57.1% (169/296)		
Asp94Asn(GAC-AAC)	10.8% (32/296)	G280A	14.5% (43/296)
Asp94Tyr(GAC-TAC)	2.7% (8/296)	G280T	
Asp94His(GAC-CAC)	0.7% (2/296)	G280C	
Asp94Ala(GAC-GCC)	14.5% (43/296)	A281C	42.9% (127/296)
Asp94Gly(GAC-GGC)	28.0% (83/296)	A281G	
Asp94Phe(GAC-TTC)	0.3% (1/296)	G280T+A281T #	
**Streptomycin**	75.8% (144/190)		

rpsL 43	69.5% (132/190)		
Leu43Ala(AAG-AGG)	69.5% (132/190)	A128G	69.5% (132/190)
rpsL 88	6.3% (12/190)		
Leu88Ala(AAG-AGG)	6.3% (12/190)	A263G	6.3% (12/190)

*, The total number of isolates when each drug is resistant; #, MM caused by 2-3 SMs at the same time.

Table S3. The details for the Occurrence of Amino Acid Missense Mutation Types between Non-XDR and preXDR/XDR Groups.

**Amino acid mutation**	**Incidence rate % (no./total no.*)**		**P value**
	**Non-XDR**	**preXDR/XDR**	
**Rifampicin**			
rpoB 511	5.6% (6/108)	2.2% (1/46)	0.618
rpoB 513	2.8% (3/108)	/	/
rpoB 516	11.1% (12/108)	8.7% (4/46)	0.872
rpoB 526	13.9% (15/108)	17.4% (8/46)	0.756
rpoB 531	65.7% (71/108)	65.2% (30/46)	1.000
rpoB 533	1.7% (2/108)	/	/
**Isoniazid**			
katG 315	60.2% (65/108)	67.4% (31/46)	0.507
inhA	19.4% (21/108)	28.3% (13/46)	0.320
**Ethambutol**			
embB 306	66.0% (117/209)	68.8% (22/32)	0.185
embB 328	1.4% (3/209)	/	/
embB 354	2.9% (6/209)	/	/
embB 406	16.3% (34/209)	15.2% (7/32)	0.450
embB 497	5.7% (12/209)	/	/
**Ofloxacin**			
gyrA 90	23.6% (64/271)	16.0% (4/25)	0.466
gyrA 91	5.2% (14/271)	8.0% (2/25)	0.634
gyrA 94	55.4% (150/271)	76.0% (19/25)	0.057
**Streptomycin**	75.2% (118/157)	78.8% (26/33)	0.824
rpsL 43	70.7% (111/157)	63.6% (21/33)	0.415
rpsL 88	4.5% (7/157)	15.2% (5/33)	0.037

* The total number of isolates when each drug is resistant; p-value < 0.05 was considered significant.
